# Signalling with retinoids in the human lung: validation of new tools for the expression study of retinoid receptors

**DOI:** 10.1186/1471-2407-9-423

**Published:** 2009-12-04

**Authors:** Stéphane Poulain, Stéphanie Lacomme, Shyue-Fang Battaglia-Hsu, Stanislas du Manoir, Lydia Brochin, Jean-Michel Vignaud, Nadine Martinet

**Affiliations:** 1 Centre de Ressources Biologiques, CHU de Nancy - Laboratoire de Pathologie Cellulaire et Moléculaire en Nutrition (LPCMN), INSERM U724, Faculté de Médecine, BP 184, 54505, Vandoeuvre-Lès-Nancy, France; 2 Institut de Génétique et de Biologie Moléculaire et Cellulaire (IGBMC), UMR 7104 CNRS-INSERM-ULP, BP 10142, 67404, Illkirch Cedex, France; 3 INSERM U728, Bât. Hayem, Hôpital Saint-Louis, 75475, Paris Cedex, France

## Abstract

**Background:**

Retinoid Receptors are involved in development and cell homeostasis. Alterations of their expressions have been observed in lung cancer. However, retinoid chemoprevention trials in populations at risk to develop such tumors have failed. Therefore, the pertinence of new clinical trials using second generation retinoid requires prior better understanding of retinoid signalling. This is our aim when validating extensively research tools, focused on *Retinoic Acid Receptor beta*, whose major role in lung cancer is documented.

**Methods:**

Biocomputing was used to assess the genomic organization of *RAR beta*. Its putative RAR-beta1' promoter features were investigated experimentally. Specific measures realized, with qRT-PCR Syber Green assays and a triplex of Taqman probes, were extensively validated to establish Retinoid Receptors mRNAs reference values for *in vivo *normal human bronchial cells, lung tumors and cell lines. Finally, a pan-RAR-beta antibody was generated and extensively validated by western-blot and immunoprecipitation.

**Results:**

No promoter-like activity was found for RAR-beta1'. *RAR-beta2 *mRNAs increase signs the normal differentiation of the human bronchial epithelium while a decrease is observed in most lung cancer cell lines. Accordingly, it is also, along with *RXR beta*, down-regulated in lung tumors. When using nuclear extracts of BEAS-2B and normal lung cells, only the RAR-beta2 long protein isoform was recognized by our antibody.

**Conclusion:**

Rigorous samples processing and extensive biocomputing, were the key factors for this study. mRNA reference values and validated tools can now be used to advance researches on retinoid signalling in the lung.

## Background

Vitamin A and its active derivatives referred to as retinoids are non-steroid hormones which play a critical role in the development and homeostasis of vertebrate tissues [1-3]. They exert their actions by regulating the expression of target genes, influencing likewise: cell proliferation, differentiation and apoptosis through nuclear Retinoid Receptors (RRs) binding [4]. Six different RRs are encoded by separate genes (*Retinoic Acid Receptors *(*RAR*) *α, β, γ *and *Retinoid X Receptors *(*RXR*) *α, β, γ*) with at least two isoforms for each, depending upon promoter usage and alternative splicing [5]. RAR/RXR heterodimers are the functional units transducing the retinoid signal when binding Retinoic Acid (RA), co-activators and DNA response elements (RAREs) of target genes [6,7]. RXRs form homodimers and/or heterodimers with RARs and most other nuclear receptors for extended effects [8,9].

Alterations in RRs expressions are observed in several cancers. The *RARα *gene is involved in acute promyelocytic leukemia [10] and is also silenced through promoter hypermethylation in human breast carcinoma cell lines [11]. In lung, breast and prostate tumors, the expression of *RARβ *is found lost or down-regulated through unconstant promoter hypermethylation, but without any deletion or mutation of this gene [12-15]. The involvement of *RARγ *in cancer is less known [16]. Down-expressions of *RXRs *have been reported in prostate and thyroid carcinoma [17,18]. *RARβ *has been mostly studied in lung cancer [19-21]. For this serious disease, primary prevention, that is tobacco eviction, remains the best goal. However, considering the overall poor results obtained by the current treatments, it seems important to offer to "at risk" subjects a secondary chemoprevention. Retinoids have been used with such a goal but have given deceiving results for still unexplained reasons [22,23]. This underlines the need for more knowledge about retinoid signalling in the normal human lung.

In this context, we recapitulated the informations available *in silico *for the human *RARβ *in order to update its genomic organization. The activity of a putative promoter RAR-beta1' was assessed experimentally. Reference values for RRs mRNAs were measured in Normal Human Bronchial Epithelial cells (NHBE) with qRT-PCR Syber Green assays and a triplex of Taqman probes. Conceived to save invaluable bronchial mucosa samples, the reliability of this last qRT-PCR tool was extensively validated. Hence, it was used to measure the relative RRs mRNAs levels in lung tumors and cell lines. Finally, contrary to commercial antibodies, our pan-RARβ antibody immunoprecipitated a single band of protein in nuclear extracts of BEAS-2B and NHBE cells. Its molecular weight and further amino acid sequencing demonstrated that it corresponds to the RARβ2 protein isoform. Rigorous samples processing, like extensive biocomputing analysis were the key factors for the realization of this study. Validated tools are now available for future investigations on Vitamin A/retinoid signalling in the lung.

## Methods

### Samples

This study was approved by the French Medical Ethical Committee for Bioresearch of Lorraine. All the samples were provided by the "Centre de Ressources Biologiques du Centre Hospitalier Universitaire de Nancy", an ISO 9001-2000 certified biobank. Informed consents were obtained from patients operated upon for lung cancer who donated their samples to the biobank. Samples collections have been extensively described in [24]. All the reagents were purchased from Sigma-Aldrich (L'Isle d'Abeau Chesnes, Saint-Quentin Fallavier, France), unless mentioned. Twelve Squamous Cell epidermoid Lung Cancer (SqCLC) samples were obtained from different patients. Fourty μm frozen sections were cut and further dissected by JMV, our pathologist, after quick staining with RNAse-free azur blue solution. Contaminating normal tissues were removed and RNA was further prepared to give 90% tumoral mRNA [24].

Trachea and the beginning of the two main bronchi were obtained from ten donors. All subjects were kept under artificial respiration until the specimens were collected. Then, samples were bathed in cold RPMI-1640 complete medium supplemented with 10% Foetal Calf Serum, 50 units/mL penicillin, 50 μg/mL streptomycin and 5 μg/mL fungizone for express transport to the laboratory. There, they were cut open and the mucosa was washed with RPMI complete medium before being scratched with the back of a scalpel blade to give "Normal differentiated Human Bronchial Epithelial cells" or NHBE: our reference tissue sample. Pooled NHBE were kept frozen at -20°C until further processing. Histological examination demonstrated that such samples contained mainly differentiated bronchial cells. The remaining scratched mucosa was dissected out from the bronchi and cut into small explants to give rise to "Cultured undifferentiated Human Bronchial Epithelial cells" (CHBE) in LH9 medium (Cambrex Bio Sciences, Emerainville, France). Cells were grown for two weeks with regular medium change as in [25]. A week later, explants were detached from dishes and cultured again twice. At confluence, CHBE were scrapped and processed like NHBE for RNA purification. Histological examination revealed mostly undifferentiated basal bronchial cells. The smooth muscle sheaths from the back of the trachea were obtained by dissection and were cut frozen as 20 μm sections. Skin samples were collected from three different female patients undergoing fat reduction and were cut also as 20 μm frozen sections parallel to the basal membrane. Quick histological examination of every third section verified that collection was restricted to epidermis. All specimens were processed as NHBE to prepare RNA.

The BEAS-2B [CRL-9609] transformed human bronchial cell line, the pleural mesothelioma NCI-H513, the Small Cell Lung Cancer NCI-H69 [HTB-119], the Non-Small Cell Lung Cancers (NSCLC) NCI-H1648 [CRL-5882], NCI-H2087 [CRL-5922] and NCI-H2342 [CRL-5941], the lung A549 [CCL-185] and the breast MDA-MB-231 [HTB-26] adenocarcinoma were purchased from the American Type Culture Collection (Rockville, MD, USA). All were propagated at 37°C in a 96% humid atmosphere with 4% CO_2 _in RPMI-1640 complete medium to confluence to prepare RNA as for CHBE.

### RNA preparation

In order to facilitate RNA solubilization, all frozen cut samples were first passed through a 23-Gauge needle. Total RNA was purified using the RNeasy Mini Kit (Qiagen, Courtaboeuf, France) with DNA digestion and integrity verification steps according to the manufacturer's instructions and our internal quality controls. Then, RNAs were diluted to a final concentration of 125 ng/μL and kept frozen at -20°C.

### cDNA synthesis

For each sample, 3 aliquots of 1 μg total RNA were reverse transcribed using the First-Strand cDNA Synthesis Kit (GE Healthcare Life Sciences, Orsay, France) according to the manufacturer's instructions. Serial dilutions of each cDNA at 1/5, 1/10, 1/50, 1/100 and 1/500 were performed.

### Drawing the human RARβ genomic region

**Figure 1:**
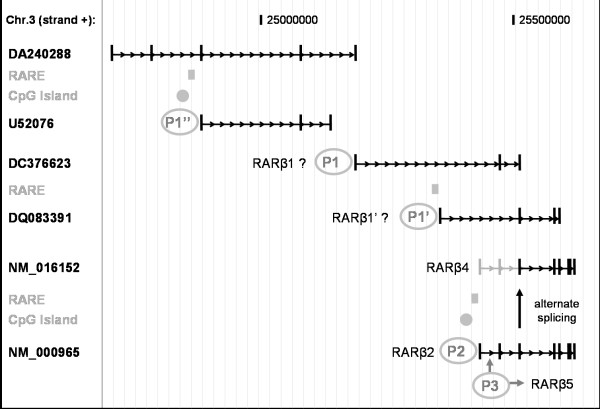
**Schematic representation of the RARβ genomic context**. Black arrows represent introns and black blocks are transcribed *RARβ *exons while grey blocks and arrows represent untranscribed spliced regions. All the promoters regions are grey circled while grey compact blocks indicate DR5 RAREs and grey rounds indicate CpG islands. P1, P1', P1", P2 and P3 indicate corresponding RAR-beta promoter regions. [GenBank:NM_000965] and [GenBank:NM_016152] are the Refseq IDs corresponding to the *RARβ *transcript isoforms 1 and 2 respectively. [GenBank:NM_000965] encodes the RARβ2 protein [Swiss-Prot:P10826-2] while [GenBank:NM_016152], resulting from the alternate splicing of [GenBank:NM_000965] at exon 3, encodes the RARβ4 protein [Swiss-Prot:P10826-3]. [GenBank:DA240288] and [GenBank:DC376623] are Expressed Sequence Tags. [GenBank:U52076] and [GenBank:DQ083391] are GenBank mRNAs. [GenBank:DC376623] and [GenBank:DQ083391] have specific first exons and share their other exons with [GenBank:NM_000965].

Genome databanks were mined to construct an extended *RARβ *genomic organization (Figure 1). Relevant PCR primers were designed to evince the presence of non validated mRNA transcripts: [GenBank:DA240288], [GenBank:U52076], [GenBank:DC376623] and [GenBank:DQ083391] (Additional File 1). The PCR program was: 94°C for 2 min, followed by 30 cycles: 94°C for 20 s, 60-64°C for 20 s, 72°C for 45 s and then 10 min at 72°C. Amplicons were analyzed by agarose gel (2%) electrophoresis. The sequences of the 5' flanking regions (-1500/-50) of each upstream transcript were further screened for promoter features with: Proscan version 1.7 [26], Promoter 2.0 [27], and Promoter Inspector [28]. Additional analysis with MatInspector [28] and TFSEARCH [29] identified transcription factors DNA binding sites. In order to evince any RARE, these sequences were also analyzed by ModelInspector [28] using a RARE DR5 weight matrix generated from the following RARE DR5 model sequence: [pu]g [g/t]tca = [ag]g [gt]tca/tga [ac]c [ct]. Finally, the orthologous conservation of the mouse RAR-beta1 and the human RAR-beta1' promoters were investigated using the Eldorado comparative genomics module [28].

### RAR-beta1' promoter analysis

Using NHBE DNA, this 1434 bp RARE containing sequence [chr3:25,355,884-25,357,317] was amplified by PCR in both forward and reverse orientations. Amplicons were sequenced for verification and then cloned into pGL3 Basic Luciferase Reporter Vectors (Promega, Charbonnières-les-Bains, France), using MluI and XhoI restriction sites. The forward PCR primers were: (F)5'-aatgcacgcgtccaattcaacgctttctgtacc-3' (R)5'-aacggctcgaggctctgtcaatggcttctcc-3', and the reverse primers were: (F)5'-aacggctcgagccaattcaacgctttctgtacc-3' (R)5'-aatgcacgcgtgctctgtcaatggcttctcc-3'. BEAS-2B and MDA-MB-231 cell lines were co-transfected in triplicate wells for 24 h and 48 h with the pGL3+RAR-beta1' plasmid constructs (firefly Luciferase) and the pRL-TK internal control reporter vector (*Renilla *Luciferase). Transfections were performed in 6-well plates using the jetPEI™ transfection reagent (Polyplus Transfection, Strasbourg, France) according to the manufacturer's instructions. The activity of the RAR-beta1' promoter was assessed by Luciferase reporter assays and carried out with the Dual-Glo™ Luciferase Assay reagent (Promega, Charbonnières-les-Bains, France) following the manufacturer's instructions. Resulting fluorescence was measured using a Victor^3 ^™ plate reader (PerkinElmer, Courtaboeuf, France) with fluorescence readings for firefly Luciferase (pGL3+P1'-RARβ) normalized to *Renilla *Luciferase (pRL-TK).

### Quantitative Real-Time PCR

The qRT-PCR primers and Taqman probes used in this study are presented in the Additional File 1. Primers for *β-Actin *were chosen in the gene 3' untranslated region as a RNA integrity control. The "RARβ" primers (exon 8) target all the *RARβ *transcripts generated from the RAR-beta2 promoter, while the "RARβ2" primers (exon 1) are specific to the *RARβ2 *transcript isoform. Primers used in Syber Green or in Taqman assays are identical for *RARα *and *RXRβ*, while they were made in different parts of the 5' end of the *RARβ *transcripts in order to cross confirm the results obtained with either the Taqman assays (primers located in the exons 6/7 junction) or with the Syber Green assays (primers located in the exon 8). No reliable primers were found for *RXRγ *(multiple single nucleotide polymorphisms). All primers, 5' FAM-RARβ-3' TAMRA and 5' Texas Red-RXRβ-3' Deep Dark Quencher-2 Taqman probes, were purchased from Eurogentec (Angers, France). The 5' VIC-RARα-3' TAMRA probe was synthesised by Applied Biosystems (Courtaboeuf, France).

For Syber Green assays, a *β-Actin *standard curve was first performed using the full cDNA cascade dilutions of each cDNA sample in triplicate and a RotorGene 6000 (Corbett Research, Labgene, Archamps, France). RRs were measured for the sole dilutions: 1/10, 1/50 and 1/100, in triplicates. Ten μL final volume contained: 4 μL of cDNA, 0.14 μL of 10 μM primers (140 nM each), 5 μL of Absolute QPCR SYBR Green Mix (Thermo Fisher Scientific, Courtaboeuf, France) and 0.72 μL of water. The PCR program was: 95°C for 15 min, followed by 50 cycles of 95°C: 10 s, 60°C: 20 s and 72°C: 20 s. Further melt curve analysis was conducted from 70°C to 95°C (0.5°C every 15 s). All amplicons were sequenced for confirmation. Taqman assays were performed for the same cDNA dilutions as above in duplicates. Twenty five μL final volume assays contained: 5 μL of cDNA, 0.5 μL of primers (200 nM each), 0.25 μL of probes (200 nM each), 12.5 μL of QuantiTect Multiplex PCR Mastermix (Qiagen, Courtaboeuf, France) and 3.75 μL of water. The PCR program began with a Taq Polymerase activation step at 95°C for 15 min, followed by 50 cycles of 94°C: 60 s and 60°C: 90 s. Assays with one probe at a time *vs *the triplex of probes were first compared in order to optimize equal final qPCR efficiencies.

Raw qRT-PCR data were collected with the RotorGene 6000 Series Software version 1.7.87. The comparative quantification approach [30] implemented within the built-in software was used to compute the second derivative of each amplification curve whose peak corresponds to the point at which the most efficient amplicon synthesis occurs. For each measure, the software determines a "Take-Off Point (TOP)" value, 80% before the peak and an "Amplification Efficiency (AE)" from the slope of the section of the curve between the TOP and the peak. For each gene and sample analyzed, all the TOP and AE values from the replicates were averaged to calculate the mean Take-Off Point (mTOP) and the mean Amplification Efficiency (mAE) values with their Standard Deviations (SD) (Additional File 2). The quality of each standard curve was assessed by its associated R^2 ^value, computed with automatic fluorescence threshold settings by the RotorGene software.

For statistical analysis, the qPCR data were first exported into the Bestkeeper^© ^version 1 software [31] to determine the most stable and common reference genes across all the samples. Validated reference genes were then combined into a Bestkeeper^© ^index for the normalization of RRs expression data. Then, the Rest-RG^© ^software [32] was used to transform the raw qPCR data into normalized RR x-fold expression ratios (R). It uses the mathematical model described by Pfaffl *et al. *[33], that combines gene quantification and normalization, according to the following equation: Ratio (R) = (E_target_)^ΔC^_target_^(control-sample)^/(E_ref_)^ΔCt^_ref_^(control-sample)^.

Ratios were computed using a Pair Wise Fixed Reallocation Randomisation Test^© ^and plotted with Standard Error (SE) estimation via a Taylor algorithm. Computations were based upon the sample-specific mAE (mAE↔E) and the corresponding mTOP deviations (ΔmTOP↔ΔCt) for each sample tested *vs *the control sample: NHBE. In each sample tested, RRs genes expressions were also normalized by the optimal combination of reference genes previously defined by Bestkeeper^©^.

### Western-Blot and Immunoprecipitation

To produce our pan-RARβ antibody, the following synthetic peptide: GHEPLTPSSSGNTAEHSPSI, corresponding to the F2 region, common to all the human RARβ proteins, was chosen to immunise rabbits and was purified by affinity chromatography (Proteogenix, Strasbourg, France). For western-blot analysis, nuclear extracts were prepared from BEAS-2B and NHBE cells as described in [34]. They were fractionated by SDS (1%) poly-acrylamide gel electrophoresis (12%) and electrotransferred onto nitrocellulose filters. After blocking with PBS containing 3% non-fat powdered milk, the filters were immunoprobed with our polyclonal RARβ antibody (dilution 1:500) for 2 h, extensively washed in PBS containing 0.05% Tween 20, and then incubated for 1 h at room temperature with an anti-rabbit peroxidase-conjugated antibody (dilution 1:40,000) (Jackson ImmunoResearch, West Grove, PE, USA). Specific complexes were revealed by chemiluminescence detection (Super Signal West Dura, Pierce Biotechnology, Rockford, IL, USA) according to the manufacturer's protocol. For immunoprecipitation analysis, NHBE nuclear extracts (1 mg) were incubated with immobilized Protein A cross-linked to the antibody, using the Seize X Protein A Immunoprecipitation Kit (Pierce Biotechnology, Rockford, IL, USA) according to the manufacturer's protocol. The immunoprecipitated proteins were detected as above by immunoblotting and chemiluminescence. Further sequencing of the immunoprecipitates were realized by the "core protein platform" of the University of Nancy.

## Results and Discussion

### Investigation of the human RARβ genomic region (Figure 1)

The different isoforms encoded by *RARβ *were first described in mice. Two murine promoters have been characterized: RAR-beta1, encoding the RARβ1 and RARβ3 proteins, and RAR-beta2, encoding RARβ2 and RARβ4 [35,36]. Then in humans, the RARβ2 protein [Swiss-Prot:P10826-2], encoded by a CpG island and RARE containing RAR-beta2 promoter region, was reported [37]. The RARβ4 protein isoform [Swiss-Prot:P10826-3], generated from the alternate splicing of the *RARβ2 *mRNA at the exon 3, was further described [5]. A human foetal protein isoform, RARβ1 [Swiss-Prot:P10826-1], also expressed in small cell lung cancers has been identified [38]. However, neither the corresponding mouse RAR-beta1-like promoter nor the *RARβ1 *and *RARβ3 *transcripts have been fully characterized in human cells. Another protein isoform, RARβ5, has been characterized in both normal and malignant human breast cells [39]. It is supposed to contribute to the resistance of the cancerous cells to retinoids. This isoform is encoded by a cryptic distinct RAR-beta3 promoter, located in the first intron of the *RARβ2 *transcript [39]. Finally, a *RARβ1' *transcript isoform, with a specific first exon, has been reported [40]. However, the corresponding protein sequence is not described. This transcript is encoded by a distinct promoter RAR-beta1" and is expressed in normal human lung and normal RA-sensitive BEAS-2B cells but it is suppressed in RA-resistant BEAS-2B, in some lung cancer cell lines and lung tumors [40].

Based upon these reports, we mined databanks to draw *in silico *the human *RARβ *genomic region (Figure 1). Two *RARβ *Refseq transcripts, [GenBank:NM_000965] and [GenBank:NM_016152], are reported on chromosome 3, strand +. They are respectively associated to the *RARβ2 *(long) and the *RARβ4 *(short) isoforms, which are transcribed from the well-characterized RAR-beta2 promoter (represented as P2 on Figure 1). These two transcripts were detected in all the samples tested when using our "RARβ" and "RARβ2" qRT-PCR primers (Additional File 1, Figure 2). No Refseq IDs were found for the *RARβ1*, *RARβ1' *and *RARβ5 *isoforms in the databanks. However, four unvalidated transcripts are reported in the region upstream of RAR-beta2: [GenBank:DQ083391], [GenBank:DC376623], [GenBank:U52076] and [GenBank:DA240288] (Figure 1). Two of these transcripts have a specific first exon in their respective sequences and share their other exons with *RARβ2 *(Figure 1). [GenBank:DC376623] is an Expressed Sequence Tag and [GenBank:DQ083391] is described as a mRNA entry that contains the partial coding sequence of *RARβ1'*. Interestingly, we found that the qRT-PCR primers used in [40] to amplify the *RARβ1' *isoform are located in the exons 1 and 2 of this unvalidated trancript. We designed specific PCR primers (Additional File 1, Figure 1) in order to establish the existence of these four upstream transcripts. However, none corresponding amplicons were found in the selected cells: NHBE, CHBE, SMC, A549, H2087, H2342 and BEAS-2B. These results were especially surprising for [GenBank:DQ083391], as the primers we designed are located in the exons 1/2 junction of the transcript and overlap with those previously used in [40] to amplify *RARβ1'*.

**Figure 2:**
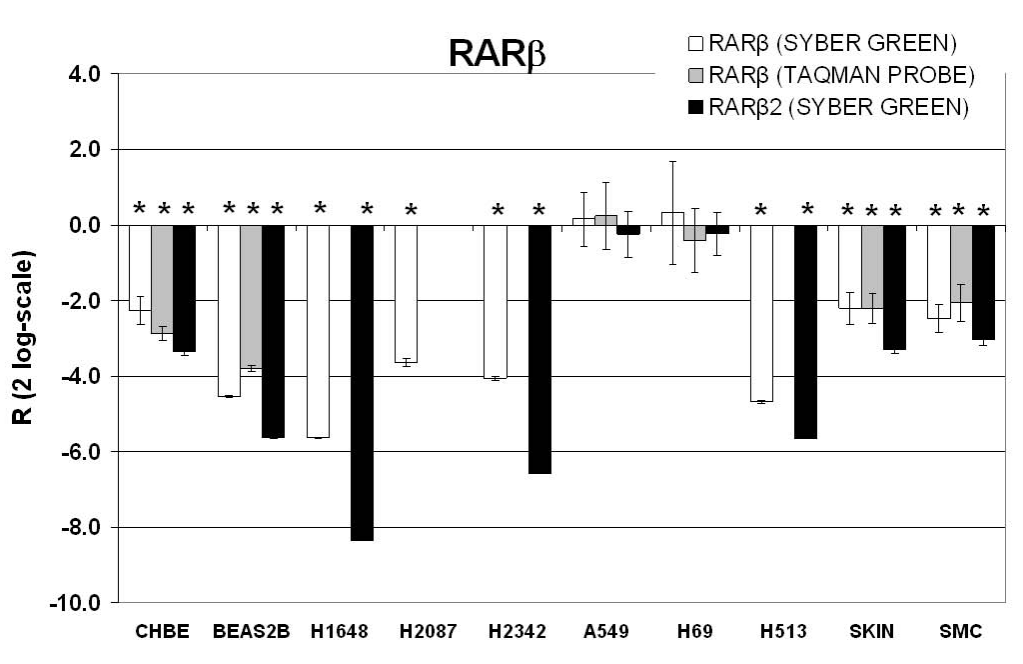
**RARβ and RARβ2 relative expression ratios**. *RARβ *x-fold ratios (R) were computed by Rest-RG^®^, based on 2-log of absolute gene regulation, with their associated standard errors. In the different samples *RARβ *expressions are normalized to reference genes and NHBE expressions. The ratios significatively different from NHBE (p-values < 0.05) are indicated with asterisks.

The upstream sequence of each unvalidated transcript (-1500/-50) was further screened using online tools to detect the features of a putative additional RAR-beta promoter region. No canonical promoter features (TATA box, ...) were found, but two degenerate DR5 RARE motifs were identified within the upstream sequences of [GenBank:U52076] and [GenBank:DQ083391], near their respective specific first exons (Figure 1). Additionally, a CpG island was found in the upstream sequence of [GenBank:U52076]. This last region (indicated as P1" on Figure 1) was previously explored by Petty *et al. *[40] and it was therefore not reinvestigated experimentally in this study. However, we searched for other possible alternate RAR-beta promoters. *In silico *comparative genomics indicated an orthologous conservation between the mouse RAR-beta1 promoter region and the upstream sequence of [GenBank:DC376623]. This region (indicated as P1 on Figure 1) corresponds to the region amplified by Houle *et al. *when cloning the 5' end of the *RARβ1 *cDNA [38]. Another interesting orthologous promoter conservation was found within the upstream sequence of [GenBank:DQ083391] for chimp *RARβ *(99%) [Genomatix:GXP_1213141] and rhesus monkey *RARβ *(95%) [Genomatix CompGen promoter]. This conserved region (indicated as P1' on Figure 1) was further investigated experimentally but no promoter-like activity was demonstrated in reporter assays. This might be a feature of weak nuclear receptors.

### Relative expressions of RRs mRNAs

Reference values for RRs mRNAs expressions were first determined in normal lung tissues (NHBE), then the relative levels of RRs mRNAs were measured in several cell lines, comparing the results obtained with the two qRT-PCR methodologies: Syber Green and a triplex of Taqman probes. The primers and probes used in this study are listed in Additional File 1. The raw qRT-PCR data are recapitulated in Additional File 2. R^2 ^values ranging from 0.965 to 1, mTOP SD<15% and mAE SD<10% demonstrates the reproducibility of each assay.

Additional File 3 shows the results obtained from the Bestkeeper^© ^analysis to select the best combination of reference genes used for the normalization of the qRT-PCR data. In addition, when demonstrating the stability of the selected genes, the quality of each sample was also systematically assessed by Bestkeeper^© ^as described in [31]. SD values < 1 demonstrates the overall inter-sample expression stability for *β-Actin *(0.56), *RARα *(0.60 with Sybr Green and 0.67 with the Taqman probe) and *RXRβ *(0.75 with Sybr Green and 0.60 with the Taqman probe). SD value > 1 sustained gene expression variability across samples and therefore exclusion from the computation of the Bestkeeper^© ^index. Using only the three stable genes, their combinations were tested to obtain the most reliable Bestkeeper^© ^index parameters. Although *RARα *correlated well with the Bestkeeper^© ^index, its inter-sample wide variation did not match with those of *β-Actin *and *RXRβ *(r < 0.5, p > 0.05). *RARα *was therefore not considered as a relevant reference gene in this case. In Syber Green assays, the optimal Bestkeeper^© ^index parameters were found for the combination of *β-Actin *and *RXRβ *as reference genes. Indeed, their expressions correlated well together (r = 0.76, p = 0.006) and a good correlation for each *β-Actin *(r = 0.96, p = 0.001) and *RXRβ *(r = 0.92, p = 0.001) was observed with the computed BestKeeper^© ^index, which exhibited a moderate SD variation of 0.64. This combination of reference genes was thus further used in each sample for the normalization of RRs expression data obtained from the Syber Green assays.

As a matter of fact, it is not possible to design a multiplex of Taqman including the housekeeping gene, *β-Actin*, and the RRs genes together because the gap of expression between them is so wide that the amplification of *β-Actin *will be inhibited when RRs become detectable for the same qPCR cDNA inputs. Since the SD values associated to the *RARα *and *RARβ *Taqman probes were > 1 (Additional File 3), they could not be used for the normalization of the data obtained with the triplex of probes. Therefore, a combination of *β-Actin *(Syber Green external reference gene) and *RXRβ *(Taqman internal reference gene) was tested. The related Bestkeeper^© ^index showed a SD variation of 0.59 (Additional File 3). The values of the selected reference genes correlated well together (r = 0.61, p = 0.046) and also with the corresponding Bestkeeper^© ^index (r = 0.94, p = 0.001 for *β-Actin *and r = 0.84, p = 0.001 for *RXRβ*). This combination of reference genes was thus used in each sample for the normalization of the data generated from Taqman assays. It also allowed to perform an accurate comparison between the significant relative expression ratios obtained with both qRT-PCR methodologies.

For each sample, the RRs mRNAs expression ratios relative to NHBE were next computed by the Rest-RG^© ^software, with a simultaneous normalization of the data by both reference genes: *β-Actin *and *RXRβ *(Additional File 4). The sample-wide relative levels of "RARβ" and "RARβ2" amplicons are represented on Figure 2. Statistical significance was determined using a Pair Wise Fixed Reallocation Randomisation Test^© ^(p-value < 0.05). We chose the method reported by Pfaffl *et al. *[33] to determine the relative expressions of RRs mRNAs because it allows a reliable normalization of the raw qRT-PCR data with an index of stable reference genes. In this method, the computation of each expression ratio is based upon the crossing point deviation (ΔCP or ΔmTOP) for each sample *vs *the control sample (NHBE). Furthermore, corrections for exact qRT-PCR efficiencies (E or mAE) are taken into account, which is not the case when computing relative expressions using the standard curve or the ΔΔCP methods [41-43]. Such normalizations are indeed very important, especially when the expressions of several genes have to be compared across numerous samples.

In Syber Green assays, the levels of "RARβ" amplicons varied as follows: H69(0.32) > A549(0.16) > skin*(-2.20) > CHBE*(-2.27) > SMC*(-2.48) > H2087*(-3.63) > H2342*(-4.06) > BEAS-2B*(-4.54) > H513*(-4.68) > H1648*(-5.63), while the levels of "RARβ2" amplicons were ranked as follows: A549(-0.24) > H69(-0.23) > SMC*(-3.03) > skin*(-3.29) > CHBE*(-3.35) > BEAS-2B*(-5.63) > H513*(-5.64) > H2342*(-6.57) > H1648*(-8.33). In H2087, the level of "RARβ2" amplicons was so weak that it was not detected. In all the samples, the relative amounts of "RARβ" amplicons were always higher than those observed for "RARβ2" (Figure 2). This is consistent with the fact that the "RARβ" primers target the common part of all the *RARβ *transcripts generated from RAR-beta2, whereas the "RARβ2" primers are specific for the sole *RARβ2 *transcript isoform. When compared to "*in vivo*" differentiated NHBE, the relative levels of both "RARβ" and "RARβ2" amplicons were always found at lower levels in the samples tested. This, especially for the "*in vitro*" undifferentiated CHBE cells, indicating that *RARβ *is indeed very important in lung cells and that an increase in *RARβ *expression signs the normal differentiation of the human bronchial epithelial tissue. In addition, the levels of "RARβ" and "RARβ2" amplicons observed in skin and SMC were very similar to CHBE and their respective amounts were also significantly lower than in NHBE. Concomitant and significant low levels of "RARβ" and "RARβ2" amplicons were observed in H1648, H2087, and H2342 (Figure 2), confirming the importance of *RARβ *in NSCLCs [21,34,44,45]. That was not the case in both H69 and A549 cell lines whose "RARβ" and "RARβ2" amplicons levels corresponded to those observed in NHBE.

The results obtained with the *RARβ *Taqman probe varied as follows: A549(0.24) > H69(-0.40) > SMC*(-2.06) > skin*(-2.21) > CHBE*(-2.87) > BEAS-2B*(-3.8). In H1648, H2087, H2342 and H513, *RARβ *was found highly down-expressed and still detected by the Syber Green assays but not by the Taqman assays (Figure 2). In order to compare the data obtained with the RARβ Taqman probe *vs *the corresponding Syber Green data, a tissue specific variation rate (VR) was computed when *RARβ *amplicons were detected in both kinds of qPCR assays and when the computed expression ratios were both significant. All the resulting VR values are < 25% (Additional File 4). This confirmed that, when detected, the *RARβ *expression ratios generated by the two qRT-PCR methodologies were consistent. The triple Taqman assay was initially developed to save rare and invaluable mRNA samples by simultaneously performing the detection of three RR genes. Since there is twice as much cDNA in Syber Green assays for a single RR amplification as there is in the Taqman probes assays to amplify three RRs, the sensitivity of the Taqman assay is therefore at least twice that of the Syber Green assay.

As *RARα*, *RARγ *and *RXRα *gave results with high SE values for all the samples (Additional File 4), their expressions were not considered significantly different from those found for NHBE. Nevertheless, in Syber Green assays, *RARα *levels are ranked as follows: skin(0.61) > SMC(0.13) > H1648(-0.02) > BEAS-2B(-0.26) > A549(-0.59) > H513(-0.64) > H2087(-0.83) > CHBE(-0.95) > H2342(-1.19) > H69(-1.30), while with the Taqman probe it is: H1648(0.10) > skin(-0.22) > SMC(-0.28) > A549(-0.31) > H513(-0.55) > BEAS-2B(-0.72) > H2087(-0.74) > CHBE(-1.35) > H2342(-1.80) > H69(-1.92). The sample-wide expression variation for *RARα *was very low, except in H69 and H2342; this confirmed the low SD values previously found for this gene when using Bestkeeper^© ^(Additional File 3). Furthermore, *RARα *expression ratios computed for Syber Green and the triplex of Taqman were consistent within each sample where *RARα *is found slightly down-expressed (H69, H2342, CHBE, H2087, H513, A549 and BEAS-2B) (Additional File 4). In skin, SMC and H1648, the *RARα *levels were similar to those observed in NHBE but the computed expression ratios varied between the qRT-PCR methodologies. The *RARγ *mRNA levels varied as follows: H513(2.27) > CHBE(1.63) > skin(1.29) > A549(0.74) > BEAS-2B(0.57) > SMC(0.53) > H2342(0.20) > H2087(-0.01) > H69(-2.32). In H1648, a very poor amplification signal was detected for *RARγ*. *RXRα *transcripts levels were ranked as follows: H513(2.05) > skin(1.53) > A549(1.14) > H1648(1.05) > CHBE(0.15) > H2342(-0.07) > SMC(-0.14) > H2087(-0.71) > BEAS-2B(-0.96) > H69(-1.75). In Syber Green assays, *RARα*, *RARγ *and *RXRα *were all found over-expressed in skin when compared to the other samples. Corresponding RRs protein levels were also previously reported as highly expressed in this tissue [46].

Significant down-expressions of *RXRs *mRNAs were previously reported in lung tumors by comparison to their respective matching normal tissues [47] and *RXRβ *was reported as a biomarker of aggressivity for NSCLCs [48]. Nevertheless, in all the lung cancer cell lines tested in this study, *RXRα *and *RXRβ *were not found differentially expressed from the control tissue NHBE. Furthermore, *RXRβ*, was demonstrated as stably expressed across all the samples, and with both qRT-PCR methodologies. In this context, it was therefore considered as a relevant reference gene.

12 dissected SqCLC tumor samples were also analyzed using *β-Actin *in Syber Green and the triplex of Taqman probes. The resulting raw qRT-PCR data are recapitulated in Additional File 5. *β-Actin *amplicons were detected in all the samples tested with Syber Green as well as *RARα *amplicons with the triplex of Taqman probes. Corresponding qRT-PCR data were analyzed with Bestkeeper^© ^to check the expression stability of the genes across samples. The results are indicated in Additional File 6. SD values < 1 demonstrated the overall expression stability for both *β-Actin *(0.89) and *RARα *(0.86) across tumoral samples. The related Bestkeeper^© ^index showed a SD variation of 0.85. The values of the selected reference genes correlated well together (r = 0.78, p = 0.003) and with the corresponding Bestkeeper^© ^index (r = 0.96, p = 0.001 for *β-Actin *and r = 0.92, p = 0.001 for *RARα*). As previously, the quality of each tumor sample was also confirmed by the software [31]. Interestingly, although *RARα *amplicons were detected for each tumor sample analyzed with the triplex of Taqman probes, no amplicons could be detected for both *RARβ *and *RXRβ *(Additional File 5). This suggests the deep concomitant down-expressions of these two RRs genes in epidermoid lung tumors.

### Western-Blot and Immunoprecipitation of RARβ

**Figure 3:**
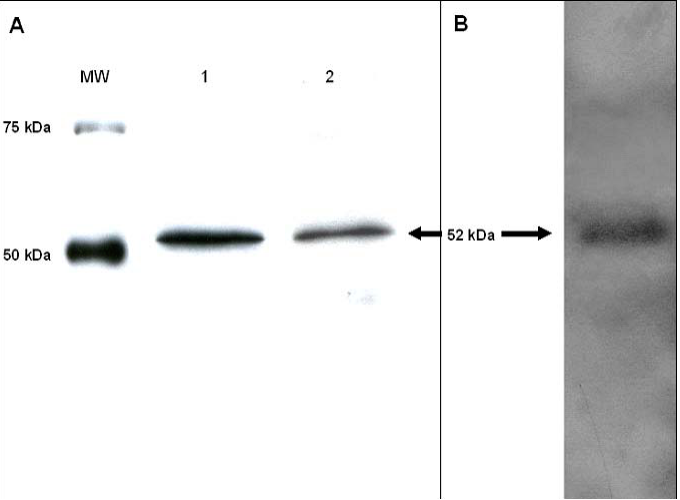
**Pan-RARβ antibody validation**. MW: Molecular Weight. Panel A: Western-Blot of BEAS-2B and NHBE nuclear extracts. lane 1: BEAS-2B nuclear extracts. lane 2: NHBE nuclear extracts. Panel B: Immunoprecipitation of BEAS-2B nuclear extracts.

Commercial antibodies against RARβ gave in our hands conflicting results in western-blots, with several protein bands that are either attributed to phosphorylation or degradation. Therefore, we validated extensively a new pan-RARβ antibody directed toward a specific peptidic sequence common to all the RARβ protein isoforms described (F2 region). Using nuclear extracts of BEAS-2B and NHBE cells, the antibody recognized a single band of about 52 kDa in both western-blot (Figure 3, panel A) and immunoprecipitation (Figure 3, panel B) experiments. The amino acid sequencing gave the following sequence: SSADHRVRLDLG (exon 5 of [NM_000965]) also common to all the RARβ proteins described, attesting for the specificity of the antibody. The results obtained correspond to the molecular weight expected for the RARβ2 protein, which is reported as a nuclear isoform of RARβ in databanks. The RARβ4 protein, whose molecular weight was previously reported at about 37 kDa [5] is reported as a cytoplasmic isoform of RARβ in normal cells [49] and it was accordingly not detected by our antibody in BEAS-2B and NHBE nuclear extracts. The RARβ1' protein isoform was previously characterized using whole cell extracts of BEAS-2B and it was reported with a molecular weight of about 47 KDa [40]. This isoform was also not detected by our antibody in the nuclear extracts analyzed. These results suggest that, if it is actually expressed in BEAS-2B and NHBE cells, the RARβ1' protein is another short cytoplasmic isoform of RARβ. That is particularly interesting as truncated isoforms of RARβ with "tumorogenic activity" were previously reported over-expressed in malignant cells [50]. Indeed, these truncated isoforms are thought to compete with RARβ2 in the nuclei of cancer cells; inhibiting its cellular functions, enhancing the proliferation process and contributing to the resistance of the cells to retinoids.

## Conclusion

In this study, we used qRT-PCR analysis to measure RRs mRNAs expressions in several samples and compared the results obtained in Syber Green assays to those given by a triplex of Taqman probes that was conceived to save invaluable mRNA samples. *RARβ *was the only RR gene whose mRNA levels were found significantly different when compared to NHBE. In these differentiated lung cells, the *RARβ *transcripts were found at their highest levels of expression, indicating that an increase in *RARβ *mRNAs signs the normal differentiation of the human bronchial epithelium. In all the samples analyzed, *RARβ2 *was the major transcript isoform that was detected. In addition, the levels of *RARβ2 *varied concomitantly with the whole levels of *RARβ *mRNAs measured in each sample. To evince the existence of additional *RARβ *isoforms, we recapitulated the informations available *in silico *for the human *RARβ*. Then, we investigated for the presence of unvalidated *RARβ *transcripts and for the activity of a putative RAR-beta1' promoter region. However, such transcripts were not detected in the samples tested and no activity was found for RAR-beta1'. This allowed to demonstrate that the whole level of *RARβ *mRNAs observed in each sample is represented by the transcripts isoforms generated from the RAR-beta2 promoter. *RARβ *transcripts were found highly down-expressed in NSCLC cell lines and SqCLC tumors, which is a reported event in the process of lung carcinogenesis. *β-Actin *and *RARα *expressions were found stable across samples. As for *RXRβ*, although its expression was found stable in normal lung cells and cancer lines, a concomitant down-regulation was observed with *RARβ *in SqCLC samples. This suggests a possible role for this RR as an heterodimerization partner for RARβ in the bronchial mucosa. Finally, RARβ2 was the only protein isoform detected by our pan-RARβ antibody in NHBE and BEAS-2B nuclear extracts, indicating that short protein isoforms of RARβ were not present within the nuclei of these cells. In our view, the research for specific human micro-RNAs targeting each of the RRs, as well as RNA interference experiments, and the over-expressions of full or truncated versions of RRs, would be of great interest in order to complete our understanding of the molecular mechanisms involved in the process of lung carcinogenesis.

## List of abbreviations

AE: Amplification Efficiency; CHBE: Cultured Human Bronchial Epithelial cells; CP: Crossing Point; DR: Direct Repeat; mAE: mean Amplification Efficiency; mTOP: mean Take-Off Point; NHBE: Normal Human Bronchial Epithelial cells; NSCLC: Non-Small Cell Lung Cancer; qRT-PCR: quantitative Real-Time Polymerase Chain Reaction; RA: Retinoic Acid; RAR: Retinoic Acid Receptor; RARE: Retinoic Acid Response Element; RR: Retinoids Receptors; RXR: Retinoid X Receptor; RXRE: Retinoid X Response Element; SCLC: Small Cell Lung Cancer; SqCLC: Squamous Cell Lung Cancer; SMC: Smooth Muscle Cell; TOP: Take-Off Point.

## Competing interests

The authors declare that they have no competing interests.

## Authors' contributions

JMV and SL were responsible for lung samples collection and preparation. NM performed cell culture of human bronchial eptithelial cells and RNA extractions. SP generated biocomputing data and performed cell culture, RNA extractions, qRT-PCR assays, promoter cloning and reporter assays. SL and LB performed RNA/DNA/Protein extractions and generated western-blot and immunoprecipitation data. SP and NM produced the manuscript. SFBH and SdM reviewed the manuscript. NM was the supervisor of this work. All authors read and approved the final manuscript.

## Authors' information

NM, senior scientist INSERM, and JMV, anathomopathologist, are the heads of the "Nancy Centre of Biological Resources" from the Teaching Hospital of Nancy, an ISO 9001-2000 certified biobank.

## Pre-publication history

The pre-publication history for this paper can be accessed here:

http://www.biomedcentral.com/1471-2407/9/423/prepub

## Supplementary Material

Additional file 1 qRT-PCR primers and Taqman probes sequences. Click here for file

Additional file 2 **Recapitulation of raw qRT-PCR data**. n: number of cDNA dilutions replicates amplified for each sample, mTOP: mean of the n Take-Off Point values computed by the Rotorgene software, mAE: mean of the n Amplification Efficiencies computed by the Rotorgene software, SD: Standard Deviation, R^2^: coefficient of correlation, Av. mAE: average of all the mAE values, ND: Not Determined. The values indicated in bold characters were further used with Bestkeeper^® ^for reference gene expression stabilty analysis. Click here for file

Additional file 3 **Bestkeeper^® ^analysis**. For each gene, the number of samples analyzed (N), the Geometric (GM) and Arithmetic Means (AM) of all the mTOP values, the maximum (Max) and minimum (Min) mTOP values are indicated with their Standard Deviations (SD [± mTOP]) and Coefficients of Variation (CV [%mTOP]). Significant SD [± mTOP] values (> 1) are indicated in bold characters. Taqman probes results are shaded in grey. The Bestkeeper indexes were computed using the combination of *β-Actin *and *RXRβ *as reference genes. For each gene, the results from the regression analysis *vs *the corresponding Bestkeeper index are indicated with their coefficients of correlation (r), coefficients of determination (r^2) and p-values. Signicant results with p-values < 0.05 are indicated in bold characters. Click here for file

Additional file 4 **Rest-RG^© ^analysis**. For each RR, the x-fold relative expression ratios (R) were computed by Rest-RG^©^, based on 2-log of absolute gene regulation, with their associated Standard Errors (SE). Ratios in the selected sample (CHBE, BEAS-2B, H1648, H2087, H2342, A549, H69, H513, skin and SMC) are normalized to reference genes and corresponding NHBE control sample expressions. Significant results with p-values < 0.05 are indicated in bold characters. Taqman probes results are shaded in grey. When the computed expression ratios are significant for both Syber Green and Taqman assays, a Variation Rate (VR) is computed to compare the results obtained with the two qRT-PCR methodologies. (ND = Not Determined). Click here for file

Additional file 5 **Recapitulation of raw qRT-PCR data computed for lung tumor samples**. n: number of cDNA dilutions replicates amplified for each sample, mTOP: mean of the n Take-Off Point values computed by the Rotorgene software, mAE: mean of the n Amplification Efficiencies computed by the Rotorgene software, SD: Standard Deviation, R^2^: coefficient of correlation, Av. mAE: average of all the mAE values, ND: Not Determined. The values indicated in bold characters were further used with Bestkeeper^® ^for reference gene expression stabilty analysis. Click here for file

Additional file 6 **Bestkeeper^® ^analysis of lung tumor samples data**. For each gene, the number of samples analyzed (N), the Geometric (GM) and Arithmetic Means (AM) of all the mTOP values, the maximum (Max) and minimum (Min) mTOP values are indicated with their Standard Deviations (SD [± mTOP]) and Coefficients of Variation (CV [%mTOP]). Significant SD [± mTOP] values (> 1) are indicated in bold characters. Taqman probes results are shaded in grey. The Bestkeeper indexes were computed using the combination of *β-Actin *and *RARα *as reference genes. For each gene, the results from the regression analysis *vs *the corresponding Bestkeeper index are indicated with their coefficients of correlation (r), coefficients of determination (r^2) and p-values. Signicant results with p-values < 0.05 are indicated in bold characters. Click here for file
