# Supplementary figures and images for: Signalling with retinoids in the human lung: validation of new tools for the expression study of retinoid receptors
Source: BMC Cancer. 2009 Dec 4;9:423. doi: 10.1186/1471-2407-9-423 (PMC2797528; doi:10.1186/1471-2407-9-423)

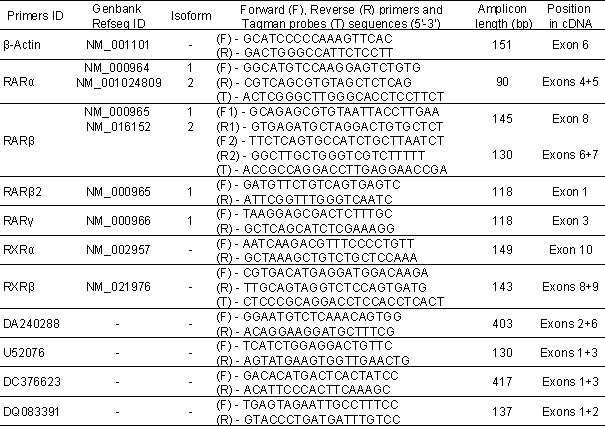

Supplement: Additional file 1 — qRT-PCR primers and Taqman probes sequences. [file 1471-2407-9-423-S1.JPEG]

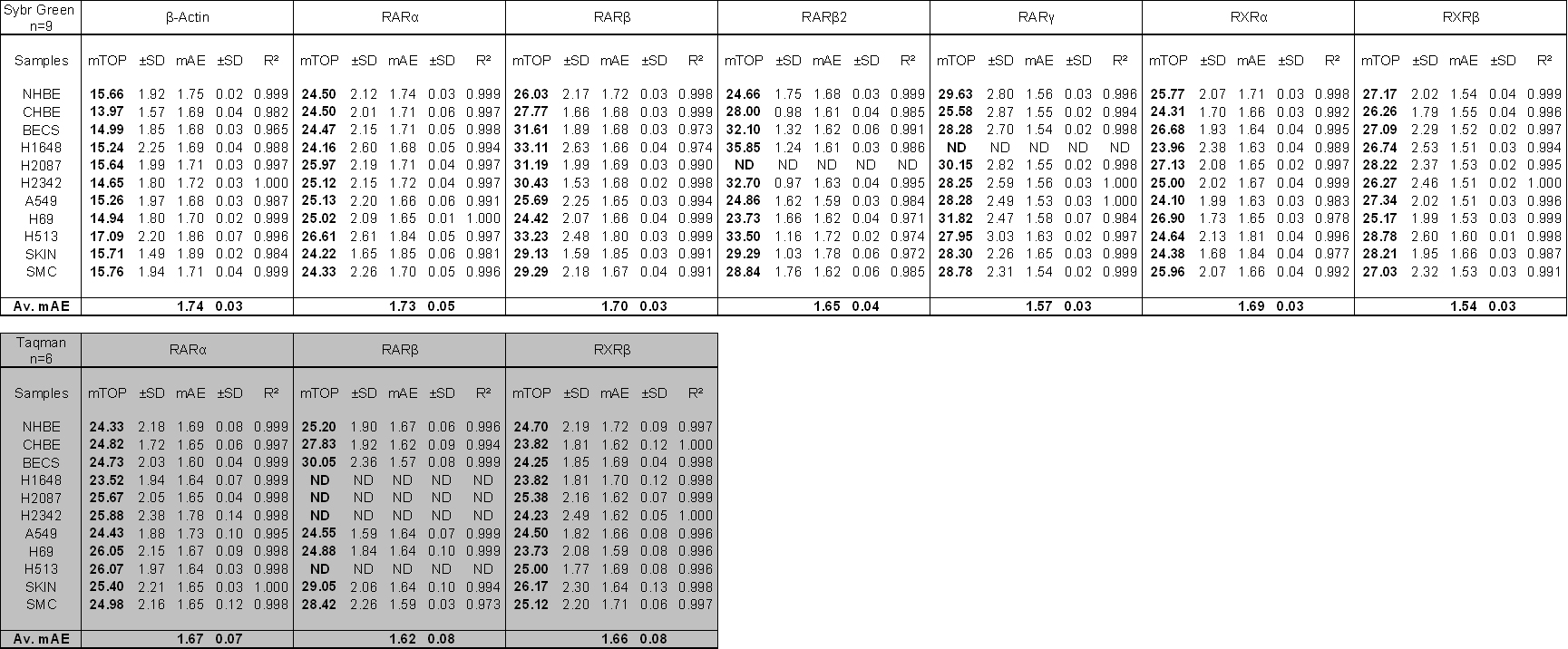

Supplement: Additional file 2 — Recapitulation of raw qRT-PCR data. n: number of cDNA dilutions replicates amplified for each sample, mTOP: mean of the n Take-Off Point values computed by the Rotorgene software, mAE: mean of the n Amplification Efficiencies computed by the Rotorgene software, SD: Standard Deviation, R2: coefficient of correlation, Av. mAE: average of all the mAE values, ND: Not Determined. The values indicated in bold characters were further used with Bestkeeper® for reference gene expression stabilty analysis. [file 1471-2407-9-423-S2.JPEG]

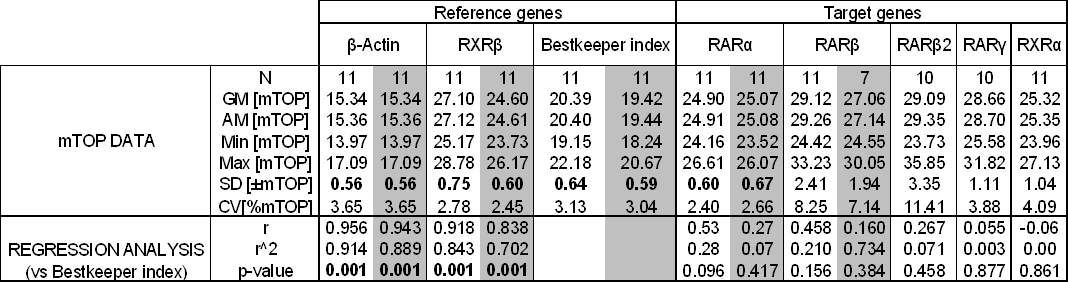

Supplement: Additional file 3 — Bestkeeper® analysis. For each gene, the number of samples analyzed (N), the Geometric (GM) and Arithmetic Means (AM) of all the mTOP values, the maximum (Max) and minimum (Min) mTOP values are indicated with their Standard Deviations (SD [± mTOP]) and Coefficients of Variation (CV [%mTOP]). Significant SD [± mTOP] values (> 1) are indicated in bold characters. Taqman probes results are shaded in grey. The Bestkeeper indexes were computed using the combination of β-Actin and RXRβ as reference genes. For each gene, the results from the regression analysis vs the corresponding Bestkeeper index are indicated with their coefficients of correlation (r), coefficients of determination (r^2) and p-values. Signicant results with p-values < 0.05 are indicated in bold characters. [file 1471-2407-9-423-S3.JPEG]

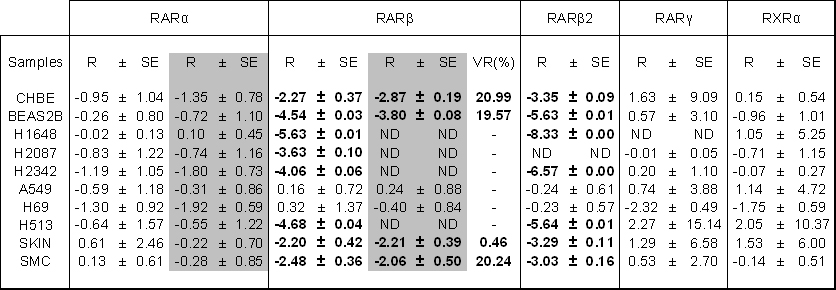

Supplement: Additional file 4 — Rest-RG© analysis. For each RR, the x-fold relative expression ratios (R) were computed by Rest-RG©, based on 2-log of absolute gene regulation, with their associated Standard Errors (SE). Ratios in the selected sample (CHBE, BEAS-2B, H1648, H2087, H2342, A549, H69, H513, skin and SMC) are normalized to reference genes and corresponding NHBE control sample expressions. Significant results with p-values < 0.05 are indicated in bold characters. Taqman probes results are shaded in grey. When the computed expression ratios are significant for both Syber Green and Taqman assays, a Variation Rate (VR) is computed to compare the results obtained with the two qRT-PCR methodologies. (ND = Not Determined). [file 1471-2407-9-423-S4.JPEG]

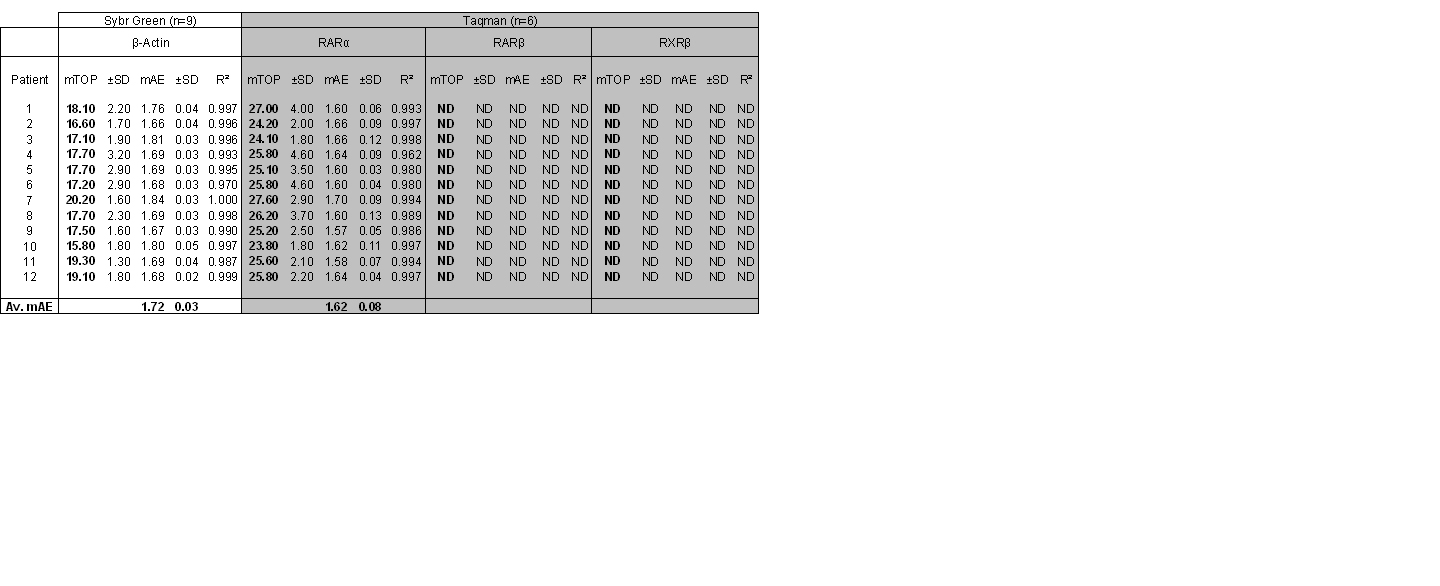

Supplement: Additional file 5 — Recapitulation of raw qRT-PCR data computed for lung tumor samples. n: number of cDNA dilutions replicates amplified for each sample, mTOP: mean of the n Take-Off Point values computed by the Rotorgene software, mAE: mean of the n Amplification Efficiencies computed by the Rotorgene software, SD: Standard Deviation, R2: coefficient of correlation, Av. mAE: average of all the mAE values, ND: Not Determined. The values indicated in bold characters were further used with Bestkeeper® for reference gene expression stabilty analysis. [file 1471-2407-9-423-S5.JPEG]

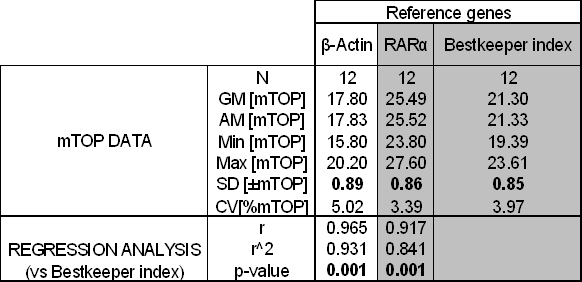

Supplement: Additional file 6 — Bestkeeper® analysis of lung tumor samples data. For each gene, the number of samples analyzed (N), the Geometric (GM) and Arithmetic Means (AM) of all the mTOP values, the maximum (Max) and minimum (Min) mTOP values are indicated with their Standard Deviations (SD [± mTOP]) and Coefficients of Variation (CV [%mTOP]). Significant SD [± mTOP] values (> 1) are indicated in bold characters. Taqman probes results are shaded in grey. The Bestkeeper indexes were computed using the combination of β-Actin and RARα as reference genes. For each gene, the results from the regression analysis vs the corresponding Bestkeeper index are indicated with their coefficients of correlation (r), coefficients of determination (r^2) and p-values. Signicant results with p-values < 0.05 are indicated in bold characters. [file 1471-2407-9-423-S6.JPEG]
